# Management of colorectal anastomotic leakage using endoscopic negative pressure therapy with or without protective ostomy: a retrospective study

**DOI:** 10.1007/s00384-021-04011-8

**Published:** 2021-08-28

**Authors:** Flavius Şandra-Petrescu, Emmanouil Tzatzarakis, Georg Kähler, Christoph Reissfelder, Florian Herrle

**Affiliations:** 1 Chirurgische Klinik, Universitätsklinikum Mannheim, Theodor-Kutzer-Ufer 1-3, 68167 Mannheim, Germany; 2 Interdisziplinäre Endoskopie, Chirurgische Klinik, Universitätsklinikum Mannheim, Theodor-Kutzer-Ufer 1-3, 68167 Mannheim, Germany

**Keywords:** Vacuum therapy, Colorectal anastomosis, Leak, Colorectal surgery, Lack of protective ostomy, Management algorithm

## Abstract

**Purpose:**

Management of colorectal anastomotic leakage (AL) is patient-oriented and requires an interdisciplinary approach. We analyzed the management of AL according to its severity and presence of ostomy and proposed a therapy algorithm.

**Methods:**

We identified all patients who underwent colorectal surgery and developed an AL in our clinic between 2012 and 2017. The management of AL was retrospectively analyzed according to the severity grade: asymptomatic (A), requesting interventional or antibiotic therapy (B), undergoing re-operation (C). The groups were compared according to the leakage characteristics, presence of ostomy, and patient clinical conditions.

**Results:**

We identified 784 consecutive patients meeting the inclusion criteria. Of these, 10.8% experienced an AL (A = 18%, B = 48%, and C = 34%). The rate of successful ostomy closure was 100% (A), 68% (B), and 62% (C), respectively. Within group B, 91% of the patients were treated solely by endoscopic negative pressure therapy (ENPT), whereas 37% of the patients within group C required ENPT in addition to surgery. Seven cases within group B (17%) required no protective ostomy (nOB) during ENPT which was itself shorter and required less cycles in comparison to group B with ostomy (OB) (*p* = 0.017 and 0.111, respectively). Moreover, the leakage distance to anal verge was higher in the OB subgroup (*p* < 0.001).

**Conclusion:**

ENPT for the treatment of colorectal AL is efficient in combination with operative revision or protective ostomy. In selected patients, it is feasible also in the absence of a protective ostomy.

## Introduction

Anastomotic leakage (AL) represents a major complication in patients undergoing colorectal surgery since it correlates with high rates of short- and long-term morbidity, as well as with increased health care costs. AL therapy depends on the intra- or extraperitoneal localization of the leak and various clinical parameters such as localized or generalized peritonitis and sepsis [1, 2]. In addition, specific conditions such as the presence of a protective ostomy, blood perfusion at the anastomotic site, or leak characteristics influence AL management. Therefore, AL management is heterogeneous, patient-oriented, and difficult to standardize. It involves non-operative as well as operative approaches or a combination of both. The former include abscess drainage, stenting or application of sealants, endoscopic negative pressure therapy (ENPT), and irrigation of the leakage cavity. The latter include anastomotic revision or redo, Hartmann’s procedure or even extirpation, and protective ostomy formation if not already present [1, 3, 4]. Despite all efforts, there is no standardized algorithm of AL therapy that is generally accepted up to date. In most of the AL cases, the first chosen approach is formation of a deviating ostomy or discontinuity resection (Hartmann’s procedure) [1]. However, protective ostomy may impair quality of life, increase postoperative morbidity due to stoma-related complications (e.g., high-output ostomy, parastomal hernia, or prolapse), prolong hospitalization, and result in permanent ostomy [5, 6]. On the other hand, ostomy reduces the clinical impact of AL and thus may allow a conservative approach such as ENPT and/or abscess drainage and help avoid an additional surgical procedure [3, 7]. The available data on AL therapy without protective ostomy is scarce. Most studies investigate AL in patients undergoing colorectal surgery with primary or secondary formation of an ostomy. Whereas ENPT of AL occurring in the upper gastrointestinal (GI) tract requires no deviation, since contamination of the leakage cavity can be avoided through a naso-jejunal feeding and a gastric decompression tube, there is a higher risk of bacterial or stool contamination as well as system blockade within the lower GI, if no protective ostomy is performed [8]. The latter may lead to a non-functional ENPT system, which may impair the anastomotic healing process or even the patient’s clinical condition. On the other hand, if ENPT without ostomy proves successful, ostomy formation may be avoided and therefore further procedures such as ostomy closure and their related morbidity could be prevented.

The aim of this study is to analyze retrospectively the management of AL by ENPT in patients undergoing colorectal surgery, with special focus on the AL therapy without protective ostomy and to propose a therapy algorithm.

## Materials and methods

### Study design and ethical approval

The present study represents a retrospective monocentric data analysis. Records from our endoscopic database, between 1 January 2012 and 31 December 2017, were analyzed for patients who have experienced AL after colorectal surgery with primary anastomosis, with or without protective ostomy. An approval of the 2^nd^ Ethics Committee of the University of Heidelberg (2019-826R) was acquired.

### Procedural management

All elective, laparoscopic, and open procedures, for malign and benign disease, were included in the analysis. In case of low anterior rectum resection with total mesorectal excision (LAR and TME) or in case of ileo- or coloanal anastomosis, a flexible silicon drain was placed adjacent to the anastomosis and was withdrawn after endoscopic control. Before the patients were discharged, a routine endoscopic control was mandatory around day 7 after index procedure for all rectal anastomoses, except the transanally hand-sewn ones. A further endoscopic control was obligatory for every anastomosis without exception before ostomy closure, or in case of clinical deterioration (e.g., fever, progressive pain, perianal bleeding), increase of serum inflammatory markers such as C-reactive protein (CRP) or CRP in combination with leukocytosis, or discharge of purulent or stool-like secretion as well as of air from the wound or the drain. In such cases, endoscopic control was often preferred to computed tomography (CT), as it provides a means of direct evaluation of the anastomosis. In case of AL, defined as “a defect of the intestinal wall integrity leading to a communication between the intra- and extraluminal compartments,” the extension of the defect was reported in relation to the lumen circumference [9]. Decision regarding the management of the AL was made while taking into consideration the patient’s clinical condition, intestine wall defect and leakage cavity extension, leakage distance to anal verge, and blood supply at the anastomotic site.

### Endoscopic approach

**Fig. 1:**
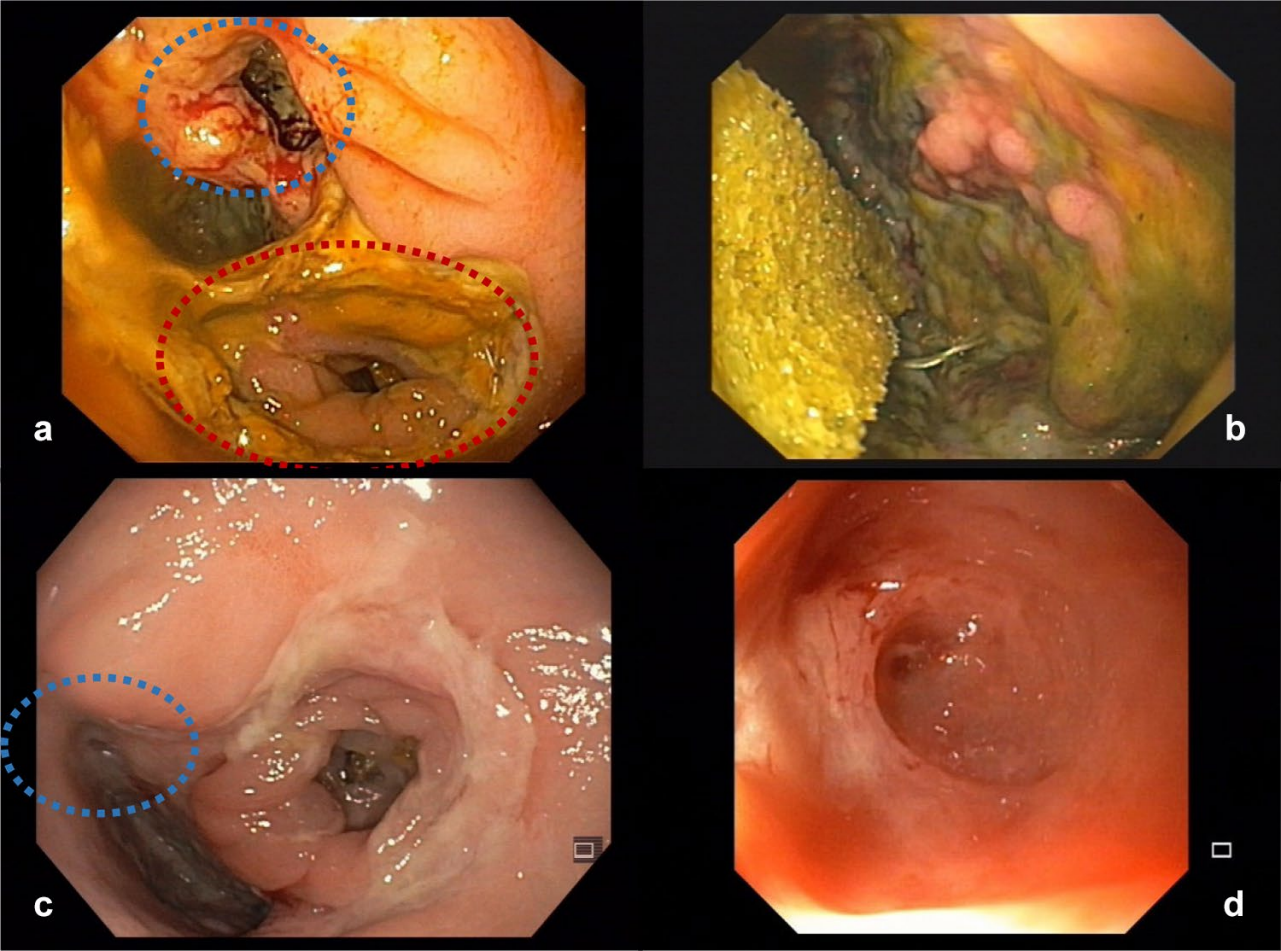
Management of grade B leak (subgroup nOB, patient no. 1) by ENPT. (**a**) AL at diagnosis (10 POD). (**b**) Endo-sponge placement into the leakage cavity. (**c**) AL at 27 POD as the ENPT was terminated (i.e., complete granulation of the leakage cavity). (**d**) Endoscopic control 3 days after ENPT completion (i.e., completely closed residual cavity). Blue circle indicates the leakage cavity, red circle indicates the colon lumen. AL, anastomotic leakage; POD, postoperative day; ENPT, endoscopic negative pressure therapy

The endoscopic therapy consisted of cavity rinse using irrigation (Endo-Technik W. Griesat, Solingen, Germany) and ENPT. The ENPT system consisted of an endo-sponge (V.A.C. Whitefoam™ Dressing, KCI Medical Products (UK), Ltd., Wimborne, Dorset, UK) that was connected with a negative pressure system (Redon – Bottle, Oriplast GmbH, Neunkirchen-Saar, Germany). Whitefoam™ Dressing is used traditionally in our clinic since it is easy to shape and no disadvantage comparing to black sponge was shown within the lower GI until now. The sponge had to fit into the leakage cavity, in order to close it completely when negative pressure was applied, and should not extend to the lumen. Thus, the entire cavity is drained and continuously downsized. Moreover, in case of stool passage, there would be minimal or no contamination of the endo-sponge or of the leakage cavity. Endoscopic changes were performed at the earliest after 3–4 days but no later than 7 days, or in case of vacuum loss or sponge dislocation. The negative pressure of the system was checked routinely every 6 h during the nurse and physician rounds. Moreover, patients were instructed to recognize the loss of negative pressure and to inform the team in charge. As the cavity shrunk, the sponge was also successively downsized. The therapy was stopped as soon as the leakage cavity was completely closed or completely covered with healthy granulation tissue (Fig. 1).

### Data processing

Patients were divided into groups according to the AL classification of the International Study Group of Rectal Cancer (ISREC), which was recently considered as the most frequently used classification [2, 9]: grade A—asymptomatic AL; grade B—AL requiring active therapy but no revision surgery; grade C—AL requiring re-laparotomy or laparoscopy. All groups were further analyzed with regard to AL management, ENPT system changes, leakage characteristics, time to cavity closure (defined as duration in days, from ENPT begin until completion), and complications. Ostomy closure or therapy completion without ostomy formation was considered as successful.

Quantitative variables are presented by median value and range. Statistical analysis was performed using Microsoft Excel and unpaired two-tailed *t*-tests (IBM SPSS Statistics 25^©^). *P* values < 0.05 were defined as statistically significant.

## Results

We identified 784 patients who underwent colorectal surgery with rectal anastomosis between 1 January 2012 and 31 December 2017 (Table 1). AL was diagnosed endoscopically in 85 cases (10.8%). Of these, 18% were grade A, 48% were grade B, and 34% were grade C; patient characteristics were similar (Table 2).

**Table 1:** Total patients undergoing colorectal surgery with anastomosis

Type of anastomosis	Descendo-rectostomy Ileo-rectostomy IPAA Total	593 (76%) 15 (2%) 176 (22%) 784
Operative approach	Laparoscopy Laparotomy Conversion Transanally*	578 (74%) 161 (21%) 40 (5%) 8 (1%)

*IPAA* ileal pouch-anal anastomosis

*3 patients underwent a combined abdominal and transanal

**Table 2:** Patient characteristics according to ISREC AL grade

AL grade (*n* = 85)	A *n* = 15 (18%)	B *n* = 41 (48%)	C *n* = 29 (34%)
Median age (range, years)	59 (28–80)	62 (31–83)	58 (19–90)
Gender (male/female)	12/3	29/12	17/12
ASA score			
I	4 (27%)	3 (7%)	4 (14%)
II	6 (40%)	27 (66%)	18 (62%)
III	5 (33%)	11 (27%)	7 (24%)
IV	0	0	0
Malign disease	8 (53%)	33 (80%)	19 (66%)
Benign disease	7 (47%)	8 (20%)	10 (34%)
Index surgery approach			
Laparoscopic	11 (73%)	19 (46%)	16 (55%)
Open	2 (13%)	12 (29%)	9 (31%)
Conversion	2 (13%)	9 (22%)	4 (14%)
Transanal	0	3 (7%)*	0
Ostomy formation during index surgery	15 (100%)	34 (83%)	29 (100%)
Median POD of AL	11	8	6
Ostomy closure	15 (100%)	23 (68%)	18 (62%)

*ASA* American Society of Anesthesiologists, *ISREC* International Study Group of Rectal Cancer, *POD* postoperative day, *AL* anastomotic leakage

*Two patients underwent combined approach, transabdominal and transanal

All patients within group A received a protective ostomy during index surgery. The ostomy closure rate within this group was 100% and no postoperative complications were observed (Table 2). Within group B, only 83% of the patients underwent colorectal resection with protective ostomy (subgroup OB). The remaining 17% (*n* = 7) received no deviation ostomy during index surgery (subgroup nOB) (Table 3). The median age within the nOB subgroup was 72 years (50–83) and all resections due to a malign disease had free margins, as confirmed by the histopathological analysis.

**Table 3:** Anastomotic leakage grade B without ostomy (subgroup nOB): patient characteristics

Patient no	1	2	3	4	5	6	7
Diagnosis	Ovarian ca. FIGO IV	Rectal/sigmoid colon ca	Rectal prolapse III°	Hartmann’s situation*	Sigmoid colon ca	Hartmann’s situation*	Rectal prolapse III°, Ogilvie sd
Age (years)	64	83	50	72	72	78	83
Gender	Female	Female	Male	Male	Male	Female	Female
ASA	II	III	I	II	II	III	II
Smoking	50 p.y	n	n	n	n	-	n
Diabetes	n	n	n	n	n	y	n
Neoadj. therapy	n	n	n	n	n	n	n
Procedure	Debulking, LAR, HE, salpingo-ovariectomy	AR + PME	Resection rectopexy	Descendo-rectostomy	Sigmoid resection, liver resection	Ileo-rectostomy	Resection rectopexy
Approach	Laparotomy	Laparotomy	Laparoscopy	Laparotomy	Laparoscopy	Laparoscopy	Laparotomy
Anastomosis	E-E stapler	E-E stapler + ventral hand-sewn	E-E stapler	E-E stapler	E-E stapler	E-E stapler	E-E stapler
TNM	pT3N1R0cM0	pT3N0R0cM0	-	-	pT3N1aR0M1 (hep)	-	-

*ASA* American Society of Anesthesiologists, *AR* anterior resection, *ca* cancer, *E-E* end to end, *FIGO* Fédération Internationale de Gynécologie et d’Obstétrique, *HE* hysterectomy, *LAR* low anterior resection, *n* no, *PME* partial mesorectal excision, *sd* syndrome, *y* yes

*Hartmann’s situation after a traumatic injury of the sigmoid colon (patient no. 4) or after subtotal colectomy due to pseudomembranous colitis (patient no. 6)

AL management within the subgroup OB was carried out mostly by ENPT (91%). The other three patients in this subgroup were treated either by CT-guided drainage of the pelvic abscess (*n* = 1), by antibiotics (*n* = 1), or by transanal drainage of the leakage cavity (*n* = 1). All patients in the subgroup nOB were treated endoscopically: ENPT (*n* = 6) and irrigation (*n* = 1). No patient within this subgroup experienced worsening of clinical condition due to AL. In one patient, anastomotic stenosis was diagnosed endoscopically and treated by dilation, and one patient experienced a membranous colitis, which was successfully treated conservatively. No patient died due to AL-related complications (Table 4).

**Table 4:** Anastomotic leakage grade B: with (OB) vs. without (nOB) ostomy

Group B (*n* = 39)	Subgroup OB (*n* = 32*)	Subgroup nOB (*n* = 7)	*p* value
POD of diagnosis (days, range)	9 (3–120)	8 (3–12)	0.363
Defect extension (in relation to lumen circumference) (range)	1/8–3/4	1/8–1/3	0.217
ENPT cycles (median) (range)	4 (1–15)	2.5 (1–8)	0.111
Frequency of endoscopic irrigations (median, range)	6 (1–17)	5 (1–8)	0.395
Therapy duration (median days) (range)	50 (8–167)	21 (4–33)	0.017
Reduced blood supply at the anastomotic site	3 (9%)**	1 (14%)	-
AL localization (cm from a.v.) (median) (range)	4.5 (2–7)	12 (5–18)	< 0.001
Parenteral nutrition	-	3 (43%)	-
High-calorie and high-protein shakes	-	6 (86%)***	-
Antibiotic therapy	28 (88%)	6 (86%)	-
Drainage of the lower pelvis during index surgery	31 (97%)	5 (71%)	-
*Two patients were excluded from the analysis as they were not treated endoluminaly; **one patient experienced a partial ischemia at the anastomotic site; ***there was no patient who received total parenteral nutrition, and in one patient, oral intake was allowed since the intestinal transit was preserved *a.v.*, anal verge; *AL*, anastomotic leakage; *ENPT*, endoscopic negative pressure therapy; *POD*, postoperative day			

*AV* anal verge, *AL* anastomotic leakage, *ENPT* endoscopic negative pressure therapy, *POD* postoperative day

*Two patients were excluded from the analysis as they were not treated endoluminaly, **one patient experienced a partial ischemia at the anastomotic site, ***there was no patient who received total parenteral nutrition, and in one patient, oral intake was allowed since the intestinal transit was preserved

**Fig. 2:**
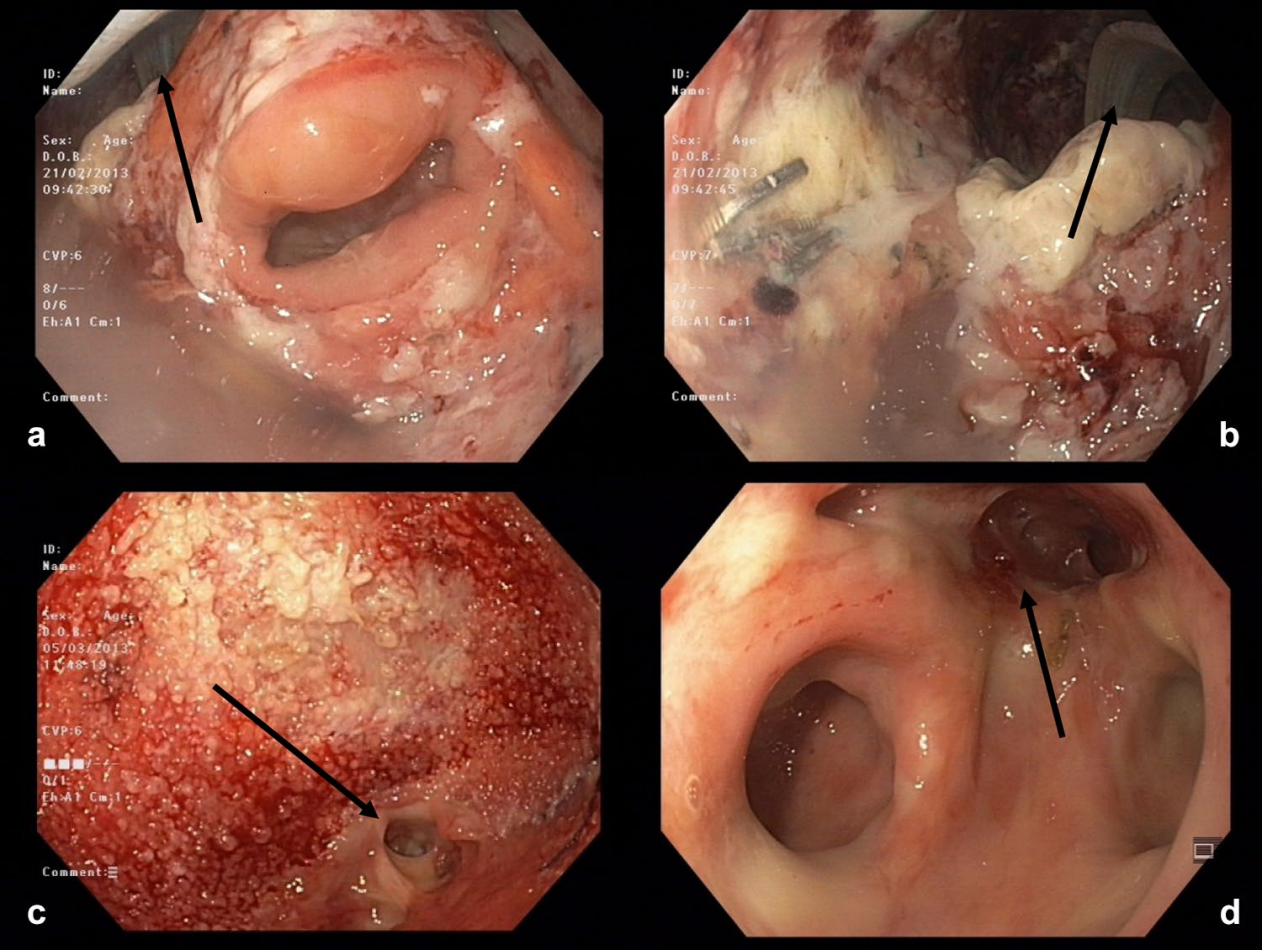
Management of grade C leak, under ostomy protection: surgical revision with laparotomy, peritoneal wash-out, perianastomotic drain (1 POD), and additional ENPT. (**a**) Almost 360° defect, colon lumen in the middle, arrow indicates the perianastomotic drain (13 POD), the cavity is still covered with fibrin. (**b**) View into the leakage cavity at 13 POD (the drain is marked with an arrow). (**c**) Leakage cavity at 25 POD, almost completely covered with granulation tissue, a persistent fistula is indicated with an arrow. (**d**) View of the anastomotic region just before ostomy closure; there is no communication with the peritoneal cavity, solely a small, blind residual cavity (indicated with an arrow), colon lumen bottom-left, blind loop bottom-right. POD, postoperative day; ENPT, endoscopic negative pressure therapy

Although all patients with AL grade C underwent surgical revision, in 38% of them, ENPT was needed additionally (Table 5) (Fig. 2).

**Table 5:** Management of AL grade C (*n* = 29)

Approach	
Laparoscopy	4 (14%)
Laparotomy	16 (55%)
Conversion	8 (28%)
Transanal	1 (3%)
Redo anastomosis	1 (3%)*
Ostomy formation	5 (17%)
Anastomotic repair (sutured repair) and ostomy	3 (10%)
Additional ENPT	11 (38%)*
Discontinuity resection (Hartmann’s procedure)	9 (31%)
Neorectal- or pouch-extirpation	2 (7%)

*AL* anastomotic leakage, *ENPT* endoscopic negative pressure therapy

*One patient underwent Hartmann’s procedure due to a recurrent anastomotic leakage

## Discussion

Despite all efforts within the last decade, incidence of colorectal AL still varies between 12 and 20% and it is therefore highly relevant for the postoperative patients’ outcome [10–13]. AL-related complications such as sepsis and localized or generalized peritonitis have a negative influence on short- and long-term outcomes, including oncological and functional ones [11, 14, 15].

AL management is heterogeneous since it depends on patient’s clinical condition and leakage characteristics. Therefore, AL therapy is patient-oriented and a generally accepted therapy algorithm is hard to be established. Based on our data, we propose a simplified AL management algorithm.

Protective ostomy reduces the clinical impact of AL in patients undergoing TAR and TME [7, 16]. Accordingly, we routinely create a protective ostomy in patients undergoing TAR and TME, as well as in patients with impaired clinical condition. This approach is also supported with strong consensus by German colorectal surgeons [7]. In our study, seven patients did not meet the previous criteria for ostomy formation (subgroup nOB) and therefore received no ostomy during index surgery. ENPT could be applied successfully in this special subgroup (nOB), since none of the patients had to undergo a secondary ostomy formation. ENPT was developed to treat complex wounds and is nowadays increasingly used for the treatment of both upper and lower GI ALs. In contrast to CT, endoscopy is of advantage since it allows a simultaneous diagnosis, characterization, and, importantly, therapy of AL [17, 18].

In this study, we identified endoscopically 85 patients with an AL among a total of 784 patients undergoing colorectal surgery with an anastomosis in the specified time period. Eighteen percent of the patients had no associated symptoms and therefore no therapy was needed (group A). All patients in this group underwent ostomy closure after 3–6 months. This suggests that spontaneous closure of the leakage cavity is expected in cases without further symptoms, as other studies have also demonstrated [1, 7]. Furthermore, 48% and 34% of the patients were included in groups B and C respectively. The rate of successful ostomy closure in these groups was 68% vs. 62%. Almost all patients in group B (92.3%) were treated by ENPT, whereas 37% of those in group C required ENPT additional to revision surgery. This emphasizes the role of endoscopic therapy in the management of AL. Several case reports with a sample size of 16 to 29 patients show a success rate of ENPT under ostomy protection of 56 to 97% [5, 19, 20]. The reported rate of ostomy closure is 20 to 88% compared to 62–68% in our study [5, 8, 20–22]. The wide range of ostomy closure rate might be explained due to the small sample size and retrospective character of the studies.

Within group B, seven patients (17%) who underwent no ostomy formation during index surgery (subgroup nOB) required no secondary ostomy formation and were treated solely by ENPT and/or endoscopic irrigation of the leakage cavity (i.e., no conversion to grade C). To our knowledge, there is little data regarding therapy of colorectal AL without protective ostomy. We identified four related case reports, none of which provides further details about the AL management or patient characteristics (Table 6).

**Table 6:** Case reports on leakage therapy without protective ostomy

study	sample size	no ostomy	secondary ostomy formation	complications	ENPT cycles (median)	therapy duration (days)(median)
Weidenhagen et al. [20]	29	8	4	sepsis, peritonitis		
Strangio et al. [22]	25	12				
von Bernstorff et al. [23]	26	8	4	sepsis	4.5	17
Arezzo et al. [24]	14	6	3	peritonitis, abscess, incompliance, no improvement of general condition	7	18

In our study, the median time to AL detection was similar in both subgroups, nOB and OB, although the range within the OB subgroup reached 120 days. This “late” leak may be explained due to the clinical condition of the patients (i.e., high ASA score, neoadjuvant radiotherapy, high Charlson Comorbidity Index) as recently suggested by van Helsdingen et al. [2]. Contrarily, “early” leaks are suggested to be more related to surgery. Every anastomosis within the subgroup nOB healed successfully after a median of 2.5 ENPT cycles in comparison to 4 cycles in the subgroup OB. The results are similar to other studies on AL therapy reporting a range of endoscopic cycles from 2.2 to 13 under ostomy protection [5, 19, 21] and lower than in studies without protective ostomy that report 4.5 or 7 cycles [23, 24]. Moreover, median duration of ENPT in the nOB subgroup is similar to previously reported data (21 vs. 17 or 18 days) [23, 24]. In contrast, the duration of therapy in subgroup OB was significantly longer. This might be explained due to an inhomogeneous patient distribution within the groups or a selection bias towards healthier patients, who were less likely to overcome the AL without needing an ostomy. However, according to our data, ENPT without ostomy does not seem to be inferior in comparison to ENPT under ostomy protection. In our study, the leakage distance from the anal verge was significantly higher and the intestine wall defect was smaller in the nOB group. However, the higher leakage localization did not lead to a peritoneal soiling as no clinical deterioration in the patients’ clinical condition or cases of generalized peritonitis were observed. Furthermore, ENPT proved successful even in the single patient in the nOB group with endoscopically observed reduced blood supply of the anastomosis. Antibiotics, which are commonly used to treat AL, were administrated similarly in both B subgroups [1]. Whether these characteristics influence the outcome of AL management without ostomy has to be further explored in prospective studies. Phitakorn et al. postulated that the size of the intestinal wall defect (< 1 cm or < 1/3 of the circumference) and of the abscess formation within the lesser pelvis abscess (< 3 cm) as well as the patient’s clinical condition should be considered when treating AL [1]. However, the study provides no information about ENPT. In our nOB subgroup, the anastomotic defect was smaller than 1/3 of the circumference. Similarly, a survey among 294 colorectal surgeons in the Netherlands argued that the anastomosis should be given a chance to heal in young and/or healthy patients (ASA I–II) [3]. Our study also included ASA III patients with stable clinical condition in both subgroups, nOB and OB. It is also of note that enteral nutrition (i.e., high-calorie and high-protein shakes) and even oral food intake in one patient did not influence the functionality of the ENPT system in our study. This may be explained due to a correct placement of the sponge into the leakage cavity.

The sponge should be placed into the deepest point of the leakage cavity and regularly tapered in order to achieve a complete collapse of the cavity. Moreover, since a constant therapy should be applied, a systematic control of the negative pressure of the system is required. All these factors reduce the risk of sponge dislocation, improving the effect of ENPT on leakage cavity downsizing. In the absence of a deviating ostomy, an intraluminal placement of the sponge may lead to an increased rate of loss of negative pressure or blockade due to stool passage. If the leakage cavity becomes too small to allow for an extraluminal sponge placement and negative pressure cannot be achieved anymore, ENPT should be removed and normal enteral nutrition may be allowed since a small, completely granulated cavity may be considered as successfully closed leakage cavity. However, the present study is a retrospective analysis of selected patients with AL undergoing ENPT without protective ostomy, and therefore further studies are required in order to prove this hypothesis.

**Fig. 3:**
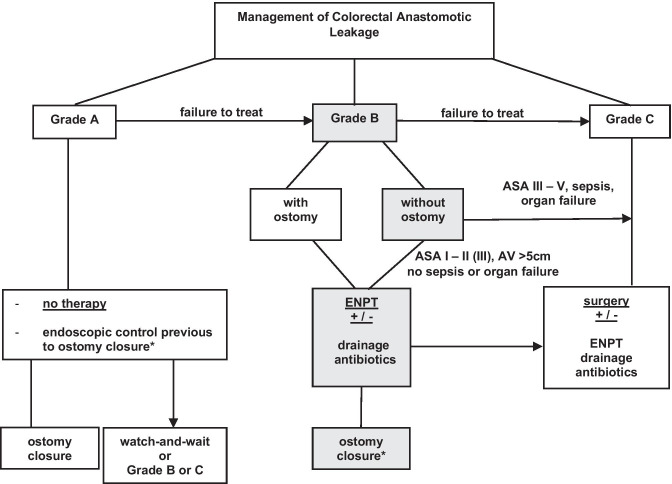
Proposal of a management algorithm of colorectal anastomotic leakage. Arrows indicate a step-up approach in case of therapy failure based on ISREC grading of leaks. ASA, American Society of Anesthesiologists; ENPT, endoscopic negative pressure therapy; ISREC, International Study Group of Rectal Cancer; AV, leakage distance to anal verge; *performed after 3 to 6 months, depending on if adjuvant therapy will be administrated or not

None of the published AL therapy algorithms to date includes the ISREC grading of AL or give any insight into the ENPT with or without ostomy [1, 3, 9]. Based on our present data, we hereby present an overview of AL management including ENPT and propose a management algorithm of AL without protective ostomy (Fig. 3). The ISREC grading of AL was developed for leakages of colorectal anastomoses after rectum resection. However, this grading is based on clinical impact of the leakage and AL was defined as “defect of the intestinal wall at the anastomotic site leading to a communication between the intra- and extraluminal compartments.” Since the definition and the clinical impact of AL do not differ, ISREC grading was applied similarly in our study, thus allowing us to retrospectively group together patients with different neorectal reservoirs who undergo the same therapy pathway based on the clinical impact of AL.

Conditions at the anastomotic site as well as the patient’s clinical condition influence the outcome of AL management. This should therefore be part of the decision-making when a therapy algorithm is chosen, in order to successfully treat an AL. Patients with no AL-related symptoms should undergo a watch-and-waiting approach, with routine endoscopic controls before ostomy closure. However, a switch to group B– or even group C–specific therapy should be considered in case of clinical deterioration.

According to our algorithm, patients with AL grade B may be treated with or without creation of an ostomy. In both situations, ENPT is an important part of the AL management and should be, according to our data, a first-line approach in the AL management. There is little data, none coming from a prospective study, on selection criteria for patients undergoing AL therapy without ostomy. Therefore, attention should be given to selecting these patients. According to our data, patients with stable clinical conditions (ASA I–II, no sepsis, no organ failure) and higher leakage distance from the anal verge (> 5 cm) should be considered as eligible for this approach. Furthermore, ASA III patients might be considered eligible if their clinical condition at the time of decision-making is stable, as evaluated by the surgeon. However, if a closure of the leakage cavity cannot be accomplished by ENPT, or in case of clinical deterioration, an escalation to grade C–specific therapy should be considered. This includes (re)ostomy, anastomotic repair or redo, Hartmann’s procedure, or even extirpation for patients with deteriorated clinical condition. Interestingly, in our study, 38% of cases within group C underwent an ENPT additional to the surgical revision in order to avoid permanent ostomy.

Avoiding of ostomy creation may reduce morbidity by eliminating complications related to the creation itself, or the future closure operation. However, patient safety is the most important criterion that has to be considered when choosing a therapy algorithm. Further unnecessary risks for patients who already experienced a major complication should be avoided. AL management should be therefore patient-oriented. The algorithm and the selection criteria suggested in our study may not be generally applicable, since they are based on retrospectively analyzed data, but could serve as a starting point for the development of a therapy algorithm for patients with AL without ostomy. Whether selection criteria and a therapy algorithm can be standardized for such a subgroup of patients has yet to be proven in further prospective studies with larger sample size. Until then, the present criteria may be considered in daily practice for patients with appropriate clinical and local conditions and whenever an ostomy formation may itself prove challenging or risky (e.g., in morbidly obese patients) as assessed by the surgeon.

## Conclusion

Based on our data, we conclude that ENPT is an effective approach in the management of colorectal anastomotic leakage grade B, which might help avoid ostomy formation in selected patients without additional morbidity. However, attention should be given to patient selection, and a step-up approach should be considered if no leakage cavity closure can be achieved or clinical deterioration occurs.
